# Quantitative nuclear phenotype signatures predict nodal disease in oral squamous cell carcinoma

**DOI:** 10.1371/journal.pone.0259529

**Published:** 2021-11-04

**Authors:** Kelly Yi Ping Liu, Sarah Yuqi Zhu, Alan Harrison, Zhao Yang Chen, Martial Guillaud, Catherine F. Poh

**Affiliations:** 1 Department of Oral Biological and Medical Sciences, Faculty of Dentistry, University of British Columbia, Vancouver, British Columbia, Canada; 2 Department of Integrative Oncology, BC Cancer Research Centre, Vancouver, British Columbia, Canada; UNITED STATES

## Abstract

**Background:**

Early-stage oral squamous cell carcinoma (OSCC) patients have a one-in-four risk of regional metastasis (LN+), which is also the most significant prognostic factor for survival. As there are no validated biomarkers for predicting LN+ in early-stage OSCC, elective neck dissection often leads to over-treatment and under-treatment. We present a machine-learning-based model using the quantitative nuclear phenotype of cancer cells from the primary tumor to predict the risk of nodal disease.

**Methods and findings:**

Tumor specimens were obtained from 35 patients diagnosed with primary OSCC and received surgery with curative intent. Of the 35 patients, 29 had well (G1) or moderately (G2) differentiated tumors, and six had poorly differentiated tumors. From each, two consecutive sections were stained for hematoxylin & eosin and Feulgen-thionin staining. The slides were scanned, and images were processed to curate nuclear morphometric features for each nucleus, measuring nuclear morphology, DNA amount, and chromatin texture/organization. The nuclei (n = 384,041) from 15 G1 and 14 G2 tumors were randomly split into 80% training and 20% test set to build the predictive model by using Random Forest (RF) analysis which give each tumor cell a score, NRS. The area under ROC curve (AUC) was 99.6% and 90.7% for the training and test sets, respectively. At the cutoff score of 0.5 as the median NRS of each region of interest (n = 481), the AUC was 95.1%. We then developed a patient-level model based on the percentage of cells with an NRS ≥ 0.5. The prediction performance showed AUC of 97.7% among the 80% (n = 23 patient) training set and with the cutoff of 61% positive cells achieved 100% sensitivity and 91.7% specificity. When applying the 61% cutoff to the 20% test set patients, the model achieved 100% accuracy.

**Conclusions:**

Our findings may have a clinical impact with an easy, accurate, and objective biomarker from routine pathology tissue, providing an unprecedented opportunity to improve neck management decisions in early-stage OSCC patients.

## Introduction

Worldwide, oral squamous cell carcinoma (OSCC) accounts for 274,000 new cases and 145,000 cancer-related deaths each year [1, 2]. Despite advances in treatment, the improvement of five-year survival rates (30–60%) remains diminutive, mainly due to the proclivity of cancer cells to spread through the lymphatics system to neck lymph nodes, which reduces survival by half [3, 4]. Therefore, neck management has been part of the treatment planning, especially for clinically node-negative necks (LN0). A commonly practiced preventative strategy is elective neck dissection (END) to remove the nodes when no clinical evidence of nodal disease is present. However, the decision of END remains subjective. Tumor depth of invasion (DOI) and differentiation are markers often used as a guide for subsequent radical neck dissection or adjuvant radiotherapy [5]. For example, DOI ≥ to 5mm has been upgraded to T2 in the recent edition of the Cancer Staging Manual of the American Joint Committee of Cancer [6]; however, DOI has been found to have limited sensitivity and specificity [7–9]. From our population-based retrospective study [10] and a pan-Canadian randomized surgical trial [11], one-in-four of the LN0 patients developed nodal disease either at the time of surgery or during post-surgery clinical follow-up. Among those who did not receive END, 25% developed nodal disease less than 12 months of surgery, and half of them deceased within less than 12 months after nodal metastasis. This infers that, if identified early, high risk clinically node negative patients may benefit from END with improved survival while others, who will not develop LN+, would avoid unnecessary neck dissection, expensive healthcare costs, prolonged hospital stays, morbidities, and adverse impact [12, 13]. Considering the significant clinical impacts, an improved objective prognostic biomarker for predicting the risk of the nodal disease is needed and can potentially guide the neck management, and consequently, reach a better survival outcome.

Quantitative pathology (QP) is a computational image analytical approach that can be used as a means to obtain objective and quantitative information concerning the diagnosis and prognosis of cancers [14]. Phenotype differences in nuclear morphology, chromatin texture, and distribution the of underlying mechanisms occurring at the genomic, transcriptomic, and epigenomic levels [15]. Our group and others have shown that differences in these phenotypes have been associated with pathologic diagnosis and progression risk across cancer types, including OSCC [16–19], prognosis [20, 21], and metastasis [22–25]. With the aid of computer and imaging technologies, QP acts as an adjunct technology that enhances the reliability, reproducibility, and capability to describe pathological changes. There is a wealth of cancer research dedicated to applying image analysis techniques to quantify microscopic features to understand the cancer pathology, diagnosis, and differential characteristics in ‘at-risk’ pre-malignant cells undergoing carcinogenic transformation. Therefore, it is conceivable that QP may also serve as a powerful tool for predicting the outcome of nodal disease.

## Objective and hypothesis

The study objective is to use a supervised machine learning method to quantify features of epithelial cancer cells that predict the risk of nodal disease. The hypothesis is that measurable nuclear morphological features of cancer cells are different between LN0 and LN+ tumors.

## Materials and methods

**Fig 1:**
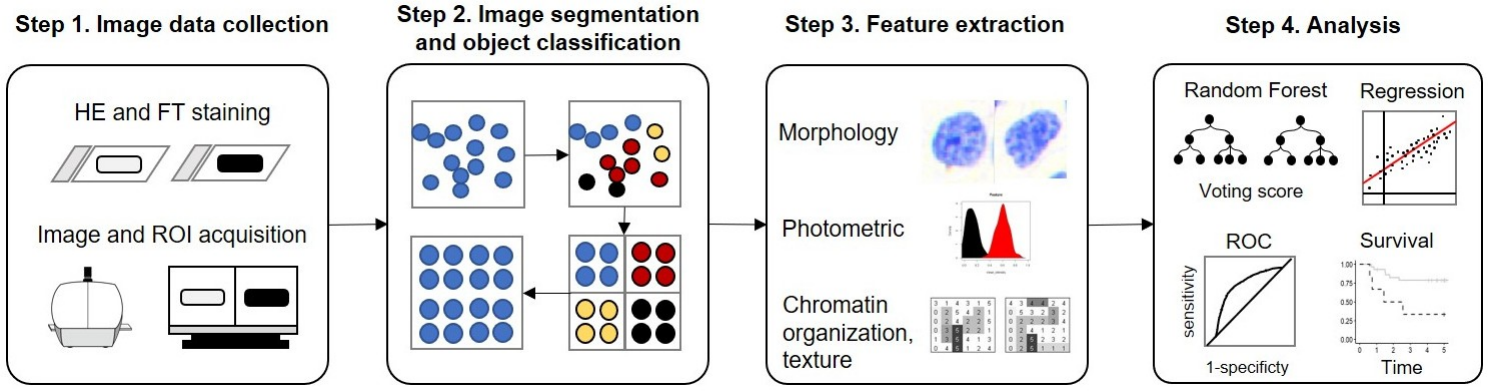
Study scheme. In Step 1, two consecutive 4-μm tissue sections stained with hematoxylin-eosin (HE) and Feulgen-thionin (FT), respectively, are scanned and reviewed by an experienced pathologist to define the region of interests (ROIs) with ~3 x 3 mm^2^. In Step 2, the images of defined ROIs are segmented to classify objects into cell groups of which features are extracted to describe nuclear morphology, photometric, and chromatin organization and texture in Step 3. In Step 4, the features are used to build a patient classification model and compared with clinical-pathological data.

The study retrospectively includes surgically resected primary tumor samples from a cohort of patients enrolled in a pan-Canadian surgical trial (NCT01039298) [11]. These patients received intent-to-cure surgery and were followed-up post-surgery for at least five years. The utilization of patient data and FFPE samples was conducted under the approval of the BC Cancer / The University of British Columbia Research Ethics Board (REB# H09-03090 and H17-02031). All patients gave written informed consent. Fig 1 illustrates the study scheme.

### Study cohort

Patients were diagnosed with OSCC from oral anatomical sites, including C02.0 to C6.0 of the ICD-10 (International Statistical Classification of Disease and Related Health Problems); clinical node-negative at the time of initial diagnosis (cLN0); enrolled in the surgical trial and received intent-to-cure surgery as the primary treatment with or without END. As a pilot study, we identified 35 primary tumors that have previously analyzed and reported [26, 27] and with enough tumor tissue for additional sections.

Outcome data included the binary status of LN+ (nodal disease confirmed by pathology) or LN0 (at the last clinical visit); time to regional recurrence (RR), which was measured from date of surgery to date of the diagnosis of nodal disease by pathology; and disease-specific survival (DSS), which was measured from the date of surgery to the date of death from OSCC. Patients who were last known to be alive and nodal-disease-free were censored at the date of the last contact.

### Sample processing and image data collection and definition of region of interests

For each of the 35 tumors, the medial tissue blocks encompassing the largest dimension of the tumor were retrieved, followed by serial sectioning into two consecutive 4-um-thick sections (S1 Fig). One slide was stained for hematoxylin and eosin (HE) staining, and the other was stained with Feulgen-thionin (FT) staining as described in the previous studies [28, 29].

The stained slides were imaged at 20x magnification on Pannoramic MIDI and reviewed on Pannoramic Viewer (3DHISTECH Ltd., Budapest, Hungary). On the HE image, the tumor areas were annotated into ~3 x 3 mm^2^ region of interests (ROIs) based on its location relative to the surface, from the surface to deep, including invasion front as the deepest 10% of total SCC layers. Given the computational limit, some ROIs were further divided into sub-areas (S1 Fig). The outlined ROIs were then extrapolated onto the corresponding FT images followed by exporting as tag image file format (TIFF) files with 1024x1024 tile size.

### Image segmentation and object classification

Each of the TIFF file was read into HistologyII, an *in-house* built program for segmentation and calculation of 93 quantitative nuclear phenotypes (QNP), which are derived from the optical density of pixels of the segmented objects [30]. The QNP describes 1) nuclear morphology, 2) photometric, and 3) chromatin organization and texture [14, 18]. The full list of QNP is described in S1 Table. After segmentation, all objects were classified into 1) good epithelial squamous, 2) good non-squamous, and 3) rejects / junk objects. S2 Fig illustrates the simplified object classification algorithm, which is a decision tree with a mixture of binary splits and Random Forests models with the input of the QNP features [31]. Once the objects are classified and cleaned, the features were normalized by the optical density of the epithelial squamous population and exported for analysis.

### Statistical analysis

Patient, tumor characteristics, and QNP features were described as either continuous or categorical variables. Comparisons between subgroups were performed by Chi-square tests for the proportion of categorical and nonparametric Wilcox rank-sum test for means of continuous variables. Given that nodal status is not a time-fixed variable, and the time of developing nodal disease during follow-up varies among the LN+ patients, the comparison of DSS between the nodal status subgroups was analyzed by using Kaplan-Meier (KM) analysis and log-rank test with a landmark time of 2-year after surgery [32]. Based on our population-based study, the majority of nodal disease events (80%) developed within two years after surgery [10]; thus, a 2-year landmark time was chosen to avoid the potential bias of neglecting patients who might have died before developing nodal disease within the 2 year. With the landmark method, patients who were alive and continued to be follow-up at 2-year were included in the KM analysis. To compare RR rates between the model predicted LN risk group, which is a time-fixed variable defined at surgery, with no deaths among the predicted negative group within 2 years, we performed by Kaplan-Meier analysis and log-rank test without the landmark method. All statistical comparisons with a P < 0.05 were considered significant. All analyses were performed using the software R (v.3.4.4) packages [33].

#### Nodal risk model development

The nodal risk score, NRS, was developed by using the Random Forests (RF) classification modeling with the input of the QNP and binary outcomes of LN+ and LN0. The RF was implemented in R using the Random Forest package [34]. To build a model to predict the nodal disease, we randomly split the cancer cells from well-differentiated (G1) and moderately differentiated (G2) tumors into 80% training and 20% test sets. The RF model was optimized for the number of trees grown from a bootstrapped sample and the number of predictors randomly tested at each node [35]. The number of trees and number of features for each node was tuned using 5-fold cross-validation, and the sample sizes were set to be equal to the smallest class to address the class imbalance issue [36]. Once the number (based on out-of-bag error rate and accuracy) of correctly classified objects are acceptable, the models were tested on the remaining 20% test set cells.

#### Predictive performance of nodal risk score

The predictive performance of NRS on LN status was assessed by using the receiver operating characteristic (ROC) curve analysis [37], and the area under the ROC curve (AUC) was used as the measure for accuracy. Based on the NRS of the training cells, a two-group cutoff value was determined to classify a cell into the LN status group, with those scored higher than the cutoff classified as ‘positive’ cells. This cutoff was then used to calculate the percentage of positive cells for each patient. To build the patient-level model, the G1/G2 patients were randomly split into 80% training and 20% test sets. ROC analysis was performed on the training patient set to determine an optimal cutoff for the percentage of positive cells that classifies a patient into risk groups, with high-risk group being patients with a percentage of positive cells greater than the cutoff. The performance of the cutoffs was then evaluated on the test patient set.

## Results

As a pilot study, a total of 35 patients, 16 LN0 and 19 LN+, were included in this study, and this includes 561 SCC ROIS with more than 468,000 cells. Table 1 summarizes the demographic and clinicopathological variables. There was no difference in age, sex, smoking history, primary tumor anatomical site, or clinical T-stage between the LN groups. As expected, poorly differentiated (G3) tumors (5 out of 6) account for most LN+ tumors. Although the depth of invasion (DOI) was significantly higher in LN+ group (9.8±6.9mm vs. 4.9±2.8mm; P = 0.01), there was no difference in terms of the DOI cutoff for END suggested by the 8^th^ edition (<5mm vs. ≥5mm: 4 vs. 15, P = 0.28). The median time to RR was 2.0 years among the 35 patients, with 6 had positive nodes at the time of surgery and 13 developed LN+ within 1.2±1.6 years after surgery. Of the 35 patients, all disease-specific deaths were experienced by LN+ patients; however, given the small sample size, the DSS rates were not statistically significant from KM analysis with a 2-year landmark time (log-rank test, P = 0.15; S3 Fig).

**Table 1:** Patient and clinical-pathological characteristics.

N (%)	Total (N = 35)	LN0 (n = 16)	LN+ (n = 19)	P value
**Age, yrs**				0.74
Mean (SD)	60.3 (16.1)	61.4 (18.2)	59.5 (14.5)	
Median (Q1, Q3)	60.2 (51.6, 71.5)	62.6 (51.3, 75.8)	58.9 (53.6, 70.0)	
**Age group**				0.25
<50	8 (22.9)	4 (25.0)	4 (21.1)	
50–72	18 (51.4)	6 (37.5)	12 (63.2)	
>72	9 (25.7)	6 (37.5)	3 (15.8)	
**Sex**				0.37
Male	19 (54.3)	10 (62.5)	9 (47.4)	
Female	16 (45.7)	6 (37.5)	10 (52.6)	
**Lesion Site Risk**				0.12
R1R2	6 (17.1)	1 (6.2)	5 (26.3)	
R3	29 (82.9)	15 (93.8)	14 (73.7)	
**Race**				0.78
Non-White	8 (22.9)	4 (25.0)	4 (21.1)	
White	27 (77.1)	12 (75.0)	15 (78.9)	
**Smoking**				0.83
Never	16 (45.7)	7 (43.8)	9 (47.4)	
Ever	19 (54.3)	9 (56.2)	10 (52.6)	
**cT**				0.17
T1	22 (62.9)	12 (75.0)	10 (52.6)	
T2	13 (37.1)	4 (25.0)	9 (47.4)	
**Grade**				0.12
G1/G2	29 (82.9)	15 (93.8)	14 (73.7)	
G3	6 (17.1)	1 (6.2)	5 (26.3)	
**DOI (mm)**				0.01
Mean (SD)	7.5 (5.9)	4.9 (2.8)	9.8 (6.9)	
Median (Q1, Q3)	6.0 (3.8, 9.5)	5.2 (1.9, 6.2)	7.0 (5.0, 14.5)	
**DOI (5mm)**				0.28
<5	10 (28.6)	6 (37.5)	4 (21.1)	
≥5	25 (71.4)	10 (62.5)	15 (78.9)	

Abbreviations: Lesion anatomical site risk (R): R1, buccal mucosa and gingiva; R2, soft palate complex; R3, tongue and floor of mouth. Clinical tumor size (cT), T1 (0–2 cm) and T1 (2–4 cm); Grade, G1, well differentiated, G2, moderately differentiated, and G3, poorly differentiated; Tumor depth of invasion (DOI), in mm, is grouped into 5mm based on the AJCC [38]; LN0, lymph node negative; LN+, lymph node positive.

### Building nodal risk score (NRS)

**Fig 2:**
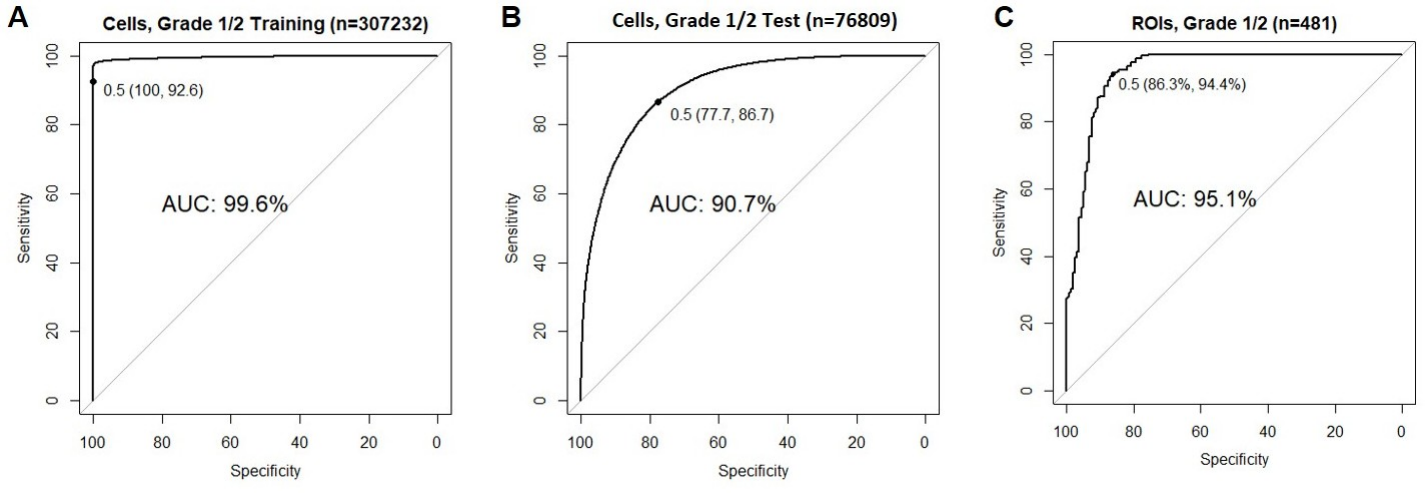
Random forest based modeled score for nodal disease. The ROC of RF-based nodal-risk score, NRS, models for training set cells (A), test set cells (B), and Grade 1 and Grade 2 ROIs (C) with the 0.5 (solid black dot) indicated as the best cutoff with the highest performance (specificity and sensitivity). The area under the curve, sensitivity, and specificity are shown in percentages. Abbreviations: NRS, nodal risk score; ROC, receiver operating characteristic curve; AUC, area under the curve; Grade 1, well differentiated tumors; G2, moderately differentiated tumors; ROIs, regions of interest; LN0, lymph node negative; LN+, lymph node positive.

**Fig 3:**
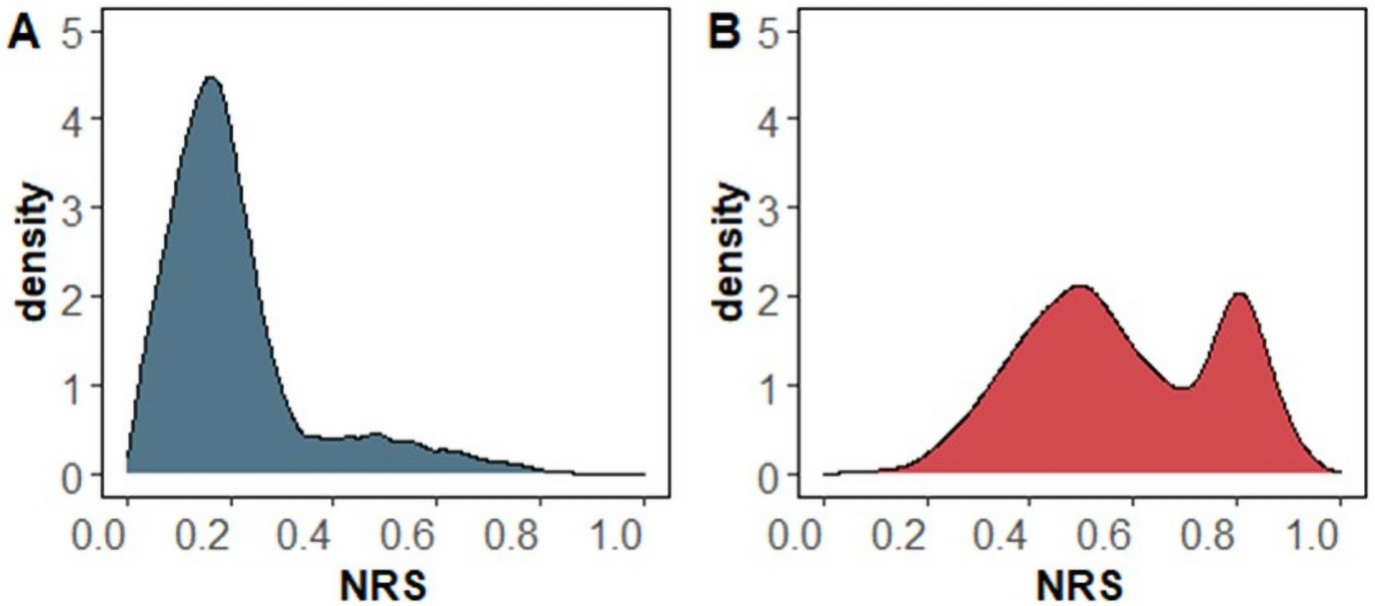
Examples of NRS distributions. The cell NRS is plotted for an (A) LN0 and (B) LN+ patient.

As aforementioned, Grade 3 (poorly differentiated) tumors are often associated with LN+, and as also observed in our dataset (5 of 6 Grade 3 were LN+), we excluded them from building the prediction model. The prediction model was built from 384,041 cells of 29 Grade 1 (well-differentiated, N = 36,156) and Grade 2 (moderately differentiated, N = 337,127) tumors. These were randomly split all cells into 80% training (N = 307,232: LN0, n = 93,687; LN+, n = 213,545) and 20% test (N = 76,809: LN0, n = 23,370; LN+, n = 53,439) sets. Two subsample sizes of the training set were set to be a similar number to avoid potential selection bias. The model, which gives each cell a score ranging from 0 to 1, was subsequently tested on the test set. Fig 2A shows the ROC curve of our model with AUC of 99.6% with an NRS of 0.5, giving us the sensitivity of 92.6% and specificity 100% (Fig 2A) for the training accuracy of 90.7% with a score of 0.5 gave us 86.7% sensitivity and 77.7% specificity for the test set (Fig 2B). Next, we assessed whether intratumor heterogeneity, the variation of the ROIs within a tumor, will impact the performance of 0.5 NRS by applying 0.5 as the cutoff across the median NRS of each ROI (n = 481: LN0, n = 161; LN+, n = 320) among the 29 G1 and G2 tumors. The AUC was 95.1, and at 0.5, the sensitivity was 86.3%, and specificity was 94.4% (Fig 2C). Examples of NRS distributions of cells are respectively shown in Fig 3A and 3B for LN0 and LN+.

### Determining optimal NRS cutoff and its predictive performance

**Fig 4:**
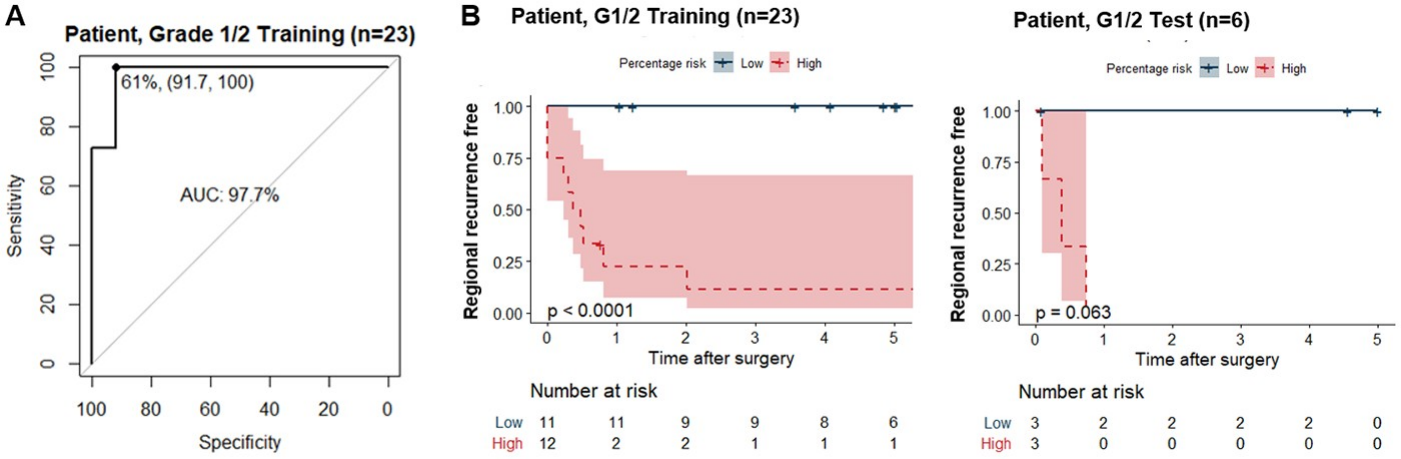
NRS predicted nodal risk. (A) Receiver operating characteristic (ROC) curve of the percentage of positive (NRS ≥ 0.5) cells among Grade 1/2 training set. The best cutoff (solid black dot) gives 100% sensitivity and 91.7% specificity. (B) Kaplan-Meier curves of regional recurrence free between low risk (blue) and high risk (red) groups of patients in the training and test sets as defined by the 61% cutoff of positive cells.

For NRS to be applicable for clinical use, we needed to build models at the patient (i.e., tumor) level. We first performed ROC analysis on each patient’s median NRS, which had AUC of 98.6% with a sensitivity 100% of and specificity of 80% at the cutoff of 0.5. We next sought to build a better model by considering the percentage of cells with ≥0.5 NRS, denoted as “positive cells”. The 29 patients were randomized into 80% training (n = 23; LN0, n = 12 and LN+, n = 11) and 20% test (n = 6; LN0, n = 3 and LN+, n = 3). There was no difference in tumor characteristics between the training and test sets (S2 Table). From the training set, the percentage of positive cells had AUC of 97.7%; and 61% was the best two-group cutoff with 100% sensitivity, 91.7% specificity, 91.7% PPV, and 100% NPV (Fig 4A) for the training set and 100% accuracy for the test set. Prediction based on the 61% outperforms other arbitrary cutoffs as summarized in S3 Table. Although the sample size was small, the predicted high-risk group showed inferior RR-free rates in both the training and the test set (log-rank test, P < 0.0001 and P = 0.06, respectively; Fig 4B).

### NRS in Grade 3 (poorly differentiated) tumor

Using the same algorithm of the percentage of positive cells on G3 tumors, a cutoff of 25% achieved the best performance with 80.0% sensitivity, 100% specificity, and 83.3% accuracy. This is comparable with tumor differentiation alone (5 of 6, 83%) when addressing Grade 3 tumors’ risk.

## Discussion

Quantitative pathology (QP) measuring nuclear phenotypic characteristics has emerged as one of the significant biomarkers informing diagnosis, treatment, and management guidance. The advantage of QP is its ability to inform nonapparent phenotypes that are consequences of underlying genetic and epigenetic alterations. Traditional pathology, such as tumor grade or DOI, is subjective and limited in the accuracy of predicting nodal disease, especially for early-stage OSCC [5, 39]. Advances in imaging analysis enable the high-throughput extraction of nuclear features to profile and assess these tumors.

The grade of differentiation is a routinely assessed phenotype based on the degree of keratinization, nuclear polymorphism, and mitosis. Poorly differentiated, Grade 3, OSCC is well recognized to be biologically more aggressive and tends to metastasize to regional lymph nodes early in the course of the disease [40]. In this study, we developed a new biomarker, the NRS, to predict nodal disease for well and moderately differentiated tumors. The rationale for such split is that Grade 3 was disproportionately higher in LN+ and Grade 1 and 2 were the majority of the cases; thus, we investigated whether a model can accurately predict Grade 1/2 tumors and whether an optimized cutoff can be applied to Grade 3 tumors with similar performance. The reported NRS model can predict nodal disease with high accuracy and can potentially serve as an adjunctive tool for clinicians’ decisions in neck management of early-stage oral cancer. When retrospectively examining our published data [10], which had 114 G3 tumors out of the 821 cases, we observed that tumor grade for the nodal disease had 73.9% accuracy and 86.0% specificity. This suggests that stratifying patients based on whether the tumor is poorly differentiated can aid in the decision of END.

The NRS provides 100% accuracy for the nodal prediction of test set of well and moderately differentiated tumors. We also observed that the model performs with similar accuracy compared to the pathology of poorly differentiation. From our previous published data [26], we found that among the 569 cases with DOI information, cutoff at 3mm, 4mm, and 5mm provide 41.9%, 46.1%, and 48.7% accuracy in predicting the nodal disease, which potentially results in overtreatment for patients with no risk of nodal disease and undertreatment for those later showing nodal disease [10]. For well and moderately differentiated tumors, the accuracy of our data is much higher than the current approach using tumor DOI.

The other innovation is the assessment of multiple ROIs within the tumor to assess the tumor heterogeneity using QNP (S4 Fig). For instance, we observed multiple modalities in the distributions of NRS among the sub-regions of some tumors. Intratumor heterogeneity represents clonal evolution and a crucial aspect in understanding the underlying evolving biology and its possible clinical implications [41, 42]. This diversity within the tumors has been an important challenge in personalized therapy as identified molecular-expression does not always represent the entire population of tumor cells [43]. As of current, there has not been in-depth research in the tumor heterogeneity of OSCC as it requires profiling of tumor at single-cell level [44–46]. Intratumoral heterogeneity is an important biology feature and can potentially impact drug therapy’s effectiveness; however, this is beyond the scope and objective of the study. Also, we did not observe its impact on the nodal risk prediction in our cases as one can see using 0.5 NRS as cutoff of the median of each ROIs, we acquire accuracy of 95.1% in predicting nodal disease.

Our group has been investigating the prognostic value of QNP in various types of cancer; however, the efforts have focused on the progression from precancer or local recurrence [18, 47]. This is the first study to use tumor-wide phenotype of OSCC and to address regional nodal metastasis, a clinically critical problem. Our results have demonstrated the superior predictive performance of the NRS. The study has a few limitations. First, quantifying nuclear features requires segmenting nuclei into complete single objects that are non-overlapping, non-touching, in-focus, and resemble the cell of interest. Based on the tumor growth patterns and behaviors, most tumors show a high proliferative index. This limits the number of well segmented objects for analysis, especially for high density areas and heavily inflamed tumors. Improved segmentation methods continue to be developed through deep-learning algorithms that could eventually bring us to maximize the number of informative objects. Second, a small sample size of tumors can be a concern; however, we have analyzed enormous data points, including >468,000 nuclei/objects and 561 ROIs. The NRS is developed via analysis of QNP of all nuclei identified. The application would be even more clinically useful when applied on small biopsy samples. To further validate the usage of 0.5 NRS, we have been prospectively collecting new independent cases for further validation.

Our study’s most important message is that prognostic and biological information enclosed in tissue can be easily acquired from a routine pathology specimen. Our data support the use of NRS as an accessible, accurate, and objective test for the decision of G1 and G2 tumors for the need of END, and poorly differentiated tumors have a high risk of nodal disease. Further validation of observed predictive performance is underway.

## Supporting information

S1 Fig Definition of region of interest. (DOCX)

S2 Fig Object segmentation and classification. All the regions of interest (ROIs) segmented into objects (i.e. nuclei) (A) which went through successive splits based on a mixture of binary or random forest algorithms (B) into populations of objects with 93 quantitative nuclear phenotypes (QNP) for analysis.
(DOCX)

S3 Fig Kaplan-Meier (KM) curve for disease-specific survival of nodal disease in with a 2-year landmark time. The curves describe 1) the survival rate of patients who either developed LN+ (red dashed curve) or LN0 (black solid curve) and were alive / followed-up by the 2-year mark (black vertical line), and 2) the survival rate of patients who continued to be followed-up pass the 2-year mark. None of the LN0 experienced death from OSCC. Abbreviation: LN0, lymph node negative; LN+, lymph node positive.
(DOCX)

S4 Fig Examples of NRS distribution among defined region of interests. (DOCX)

S1 Table Quantitative tissue phenotypes. (DOCX)

S2 Table Tumor characteristics of Grade 1/2 training and test sets. (DOCX)

S3 Table Diagnostic performance of percentage of positive cells. (DOCX)

S4 Table Nodal risk score dataset. (DOCX)
