# Supplementary material for: Quantitative nuclear phenotype signatures predict nodal disease in oral squamous cell carcinoma
Source: PLoS One. 2021 Nov 4;16(11):e0259529. doi: 10.1371/journal.pone.0259529 (PMC8568158; doi:10.1371/journal.pone.0259529)
Supplement: S1 Fig — (DOCX) [file pone.0259529.s001.docx]

| 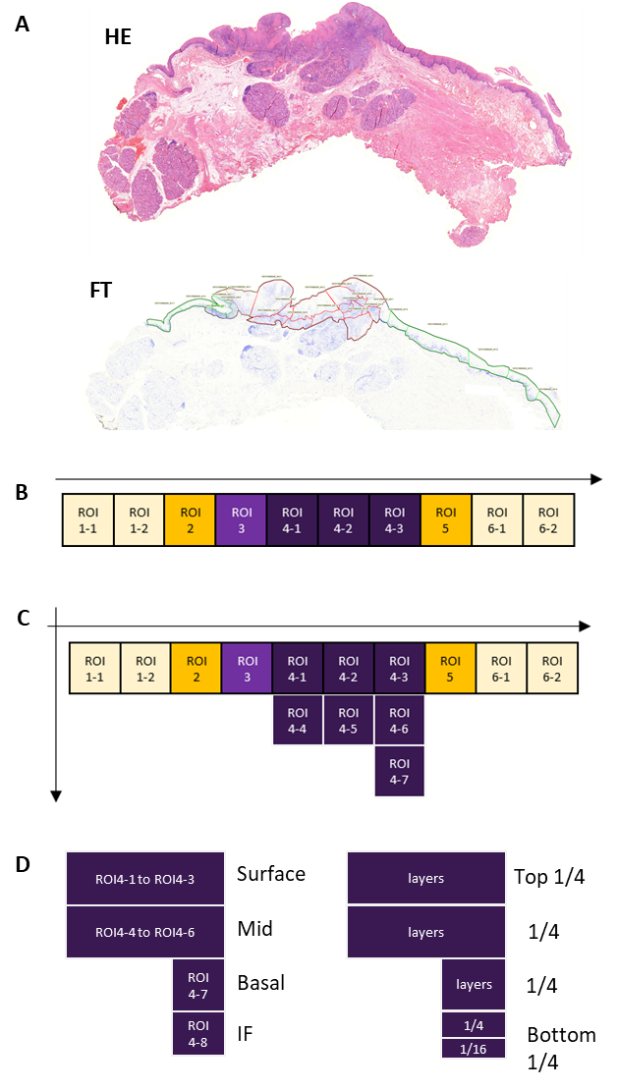 |
| --- |
| **S1 Fig. Definition of regions of interest.**  Abbreviations: HE, hematoxylin & eosin; FT, Feulgen-thionin; ROI, region of interest |
