# Supplementary material for: Quantitative nuclear phenotype signatures predict nodal disease in oral squamous cell carcinoma
Source: PLoS One. 2021 Nov 4;16(11):e0259529. doi: 10.1371/journal.pone.0259529 (PMC8568158; doi:10.1371/journal.pone.0259529)
Supplement: S2 Fig — All the regions of interest (ROIs) segmented into objects (i.e. nuclei) (A) which went through successive splits based on a mixture of binary or random forest algorithms (B) into populations of objects with 93 quantitative nuclear phenotypes (QNP) for analysis. (DOCX) [file pone.0259529.s002.docx]

| 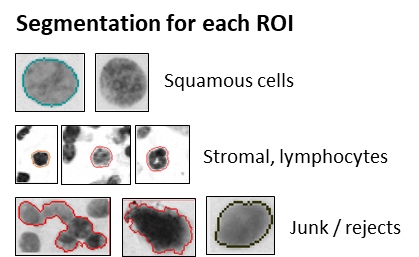 | 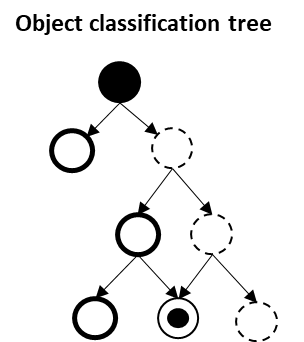 |
| --- | --- |
| ● All segmented objects (~8000 to 15,000 per ROI) | |
| 🞆 Good epithelial squamous objects | |
| 🞊 Good non-squamous objects (i.e. stromal) | |
| ◌ Rejects / junk | |

**S2 Fig. Object segmentation and classification.** All region of interests (ROIs) are segmented into objects (i.e. nuclei) (A) which went through successive splits based on a mixture of binary or random forest algorithms (B) into populations of objects with 93 quantitative nuclear phenotypes (QNP) for analysis.
