# Supplementary material for: Quantitative nuclear phenotype signatures predict nodal disease in oral squamous cell carcinoma
Source: PLoS One. 2021 Nov 4;16(11):e0259529. doi: 10.1371/journal.pone.0259529 (PMC8568158; doi:10.1371/journal.pone.0259529)
Supplement: S3 Fig — The curves describe 1) the survival rate of patients who either developed LN+ (red dashed curve) or LN0 (black solid curve) and were alive / followed-up by the 2-year mark (black vertical line), and 2) the survival rate of patients who continued to be followed-up pass the 2-year mark. None of the LN0 experienced death from OSCC. Abbreviation: LN0, lymph node negative; LN+, lymph node positive. (DOCX) [file pone.0259529.s003.docx]

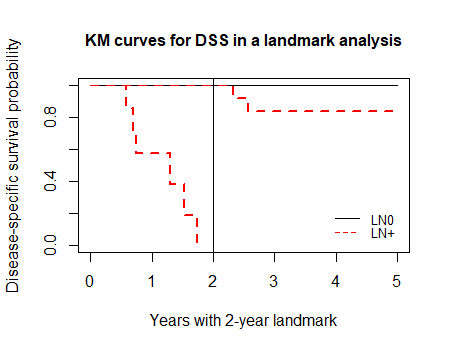


**S3 Fig.** Kaplan-Meier (KM) curve for disease-specific survival of nodal disease in a with a 2-year landmark time. The curves describe 1) the survival rate of patients who either developed LN+ (red dashed curve) or LN0 (black solid curve) and were alive / followed-up by the 2-year mark (black vertical line), and 2) the survival rate of patients who continued to be followed-up pass the 2-year mark. None of the LN0 experienced death from OSCC. Abbreviation: LN0, lymph node negative; LN+, lymph node positive.
