# Supplementary figures and images for: Quantitative nuclear phenotype signatures predict nodal disease in oral squamous cell carcinoma
Source: PLoS One. 2021 Nov 4;16(11):e0259529. doi: 10.1371/journal.pone.0259529 (PMC8568158; doi:10.1371/journal.pone.0259529)

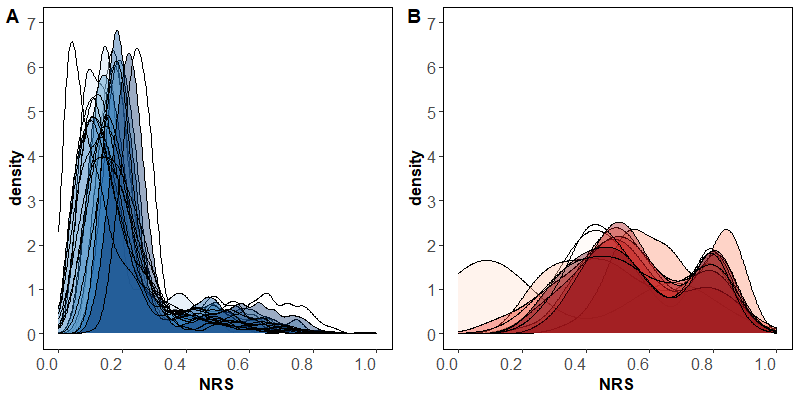


**S4 Fig. Examples of NRS distribution among defined region of interests**

Supplement: S4 Fig — (DOCX) [file pone.0259529.s004.docx]
