# Supplementary material for: Quantitative nuclear phenotype signatures predict nodal disease in oral squamous cell carcinoma
Source: PLoS One. 2021 Nov 4;16(11):e0259529. doi: 10.1371/journal.pone.0259529 (PMC8568158; doi:10.1371/journal.pone.0259529)
Supplement: S1 Table — (DOCX) [file pone.0259529.s005.docx]

**S1 Table. Quantitative nuclear phenotypes**

| **Morphology** | **Photometric** | **Discrete** | **Markovian** | **Fractal** | **Runlength_1** | **runlength_2** |
| --- | --- | --- | --- | --- | --- | --- |
| Area_m | OD_maximum | lowDNAArea | Entropy | Fractal_dimen | short0_runs | short_runs1 |
| Mean_radius_m | OD_variance | medDNAArea | Energy | Fractal1_area | short45_runs | short_runs2 |
| Max_radius_m | OD_skewness | hiDNAArea | Correlation | Fractal2_area | short90_runs | short_runs3 |
| Var_radius | OD_kurtosis | lowDNAAmnt | Contrast |  | short135_runs | short_runs4 |
| Sphericity | Mean_intensity | medDNAAmnt | homogeneity |  | long0_runs | long_runs1 |
| Eccentricity | Var_intensity | hiDNAAmnt |  |  | long45_runs | long_runs2 |
| Inertia_shape |  | lowDNAcomp |  |  | long90_runs | long_runs3 |
| Compactness |  | medDNAcomp |  |  | long135_runs | long_runs4 |
| Elongation |  | hiDNAcomp |  |  | gray0_level | gray_level1 |
| Freq_low_fft |  | mhDNAcomp |  |  | gray45_level | gray_level2 |
| Freq_high_fft |  | low_av_dst |  |  | gray90_level | gray_level3 |
| Harmon01_fft |  | med_av_dst |  |  | gray135_level | gray_level4 |
| Harmon02_fft |  | hi_av_dst |  |  | run0_length | run_length1 |
| Harmon03_fft |  | mh_av_dst |  |  | run45_length | run_length2 |
| Harmon04_fft |  | lowVSmed_DNA |  |  | run90_length | run_length3 |
| Harmon05_fft |  | lowVShigh_DNA |  |  | run135_length | run_length4 |
|  |  | lowVSmh_DNA |  |  | run0_percent | run_percent1 |
|  |  | low_den_obj |  |  | run45_percent | run_percent2 |
|  |  | med_den_obj |  |  | run90_percent | run_percent3 |
|  |  | high_den_obj |  |  | run135_percent | run_percent4 |
|  |  | low_cntr_mass |  |  |  |  |
|  |  | med_cntr_mass |  |  |  |  |
|  |  | high_cntr_mass |  |  |  |  |
|  |  |  |  |  |  |  |
|  |  |  |  |  |  |  |
