# Supplementary material for: Quantitative nuclear phenotype signatures predict nodal disease in oral squamous cell carcinoma
Source: PLoS One. 2021 Nov 4;16(11):e0259529. doi: 10.1371/journal.pone.0259529 (PMC8568158; doi:10.1371/journal.pone.0259529)
Supplement: S2 Table — (DOCX) [file pone.0259529.s006.docx]

**S2 Table. Tumor characteristics of Grade 1/2 training and test sets**

| **n (%)** | **Training (n=23)** | **Test (n=6)** | **Total (n=29)** | **P value** |
| --- | --- | --- | --- | --- |
| **Grade** |  |  |  | 0.785 |
| G1 | 5 (21.7) | 1 (16.7) | 6 (20.7) |  |
| G2 | 18 (78.3) | 5 (83.3) | 23 (79.3) |  |
| **DOI** |  |  |  | 0.938 |
| Mean (SD) | 6.513 (5.123) | 6.333 (4.274) | 6.476 (4.887) |  |
| **DOI (5 mm)** |  |  |  | 0.393 |
| <5 | 8 (34.8) | 1 (16.7) | 9 (31.0) |  |
| ≥5 | 15 (65.2) | 5 (83.3) | 20 (69.0) |  |

Abbreviations: NRS, nodal risk score; G1, well differentiated tumor; G2, moderately differentiated tumor; DOI, depth of invasion
