# Supplementary material for: Quantitative nuclear phenotype signatures predict nodal disease in oral squamous cell carcinoma
Source: PLoS One. 2021 Nov 4;16(11):e0259529. doi: 10.1371/journal.pone.0259529 (PMC8568158; doi:10.1371/journal.pone.0259529)
Supplement: S3 Table — (DOCX) [file pone.0259529.s007.docx]

**S3 Table. Diagnostic performance of percentage of positive cells^a^**

|  | **25% of positive cells** | | | | **50% of positive cells** | | | | **75% of positive cells** | | | |
| --- | --- | --- | --- | --- | --- | --- | --- | --- | --- | --- | --- | --- |
|  | **Training** | | **Test** | | **Training** | | **Test** | | **Training** | | **Test** | |
|  | **LN0** | **LN+** | **LN0** | **LN+** | **LN0** | **LN+** | **LN0** | **LN+** | **LN0** | **LN+** | **LN0** | **LN+** |
| **Low risk** | 9 | 0 | 2 | 0 | 9 | 0 | 3 | 0 | 12 | 3 | 3 | 0 |
| **High risk** | 3 | 11 | 1 | 3 | 3 | 11 | 0 | 3 | 0 | 8 | 0 | 3 |
| **Acc** | 0.87 | | 0.83 | | 0.87 | | 1.0 | | 0.87 | | 1.0 | |
| **Sensitivity** | 1.0 | | 1.0 | | 1.0 | | 1.0 | | 0.72 | | 1.0 | |
| **Specificity** | 0.75 | | 0.67 | | 0.75 | | 1.0 | | 1.0 | | 1.0 | |
| **PPV** | 0.79 | | 0.75 | | 0.79 | | 1.0 | | 1.0 | | 1.0 | |
| **NPV** | 1 | | 1,0 | | 1.0 | | 1.0 | | 0.8 | | 1.0 | |

^a^cells with NRS greater or equal to 0.5

Abbreviations: NRS, nodal risk score; LN0, lymph node negative; LN+, lymph node positive; Acc; accuracy, PPV, positive predictive value; NPV, negative predictive value
