# Supplementary material for: Quantitative nuclear phenotype signatures predict nodal disease in oral squamous cell carcinoma
Source: PLoS One. 2021 Nov 4;16(11):e0259529. doi: 10.1371/journal.pone.0259529 (PMC8568158; doi:10.1371/journal.pone.0259529)
Supplement: S4 Table — (DOCX) [file pone.0259529.s008.docx]

**S4 Table. Nodal risk score dataset**

| Patient identifier | LN | Grade | DOI | NRS_mean | NRS_sd | NRS_median | cells_negative | cell_positive | | Positive% |
| --- | --- | --- | --- | --- | --- | --- | --- | --- | --- | --- |
| C025 | 0 | G2 | 2 | 0.177 | 0.164 | 0.154 | 4856 | 325 | | 6.27 |
| C031 | 1 | G2 | 5 | 0.783 | 0.141 | 0.814 | 598 | 13263 | | 95.69 |
| V008 | 0 | G2 | 11 | 0.210 | 0.146 | 0.171 | 5255 | 376 | | 6.68 |
| V013 | 0 | G1 | 1.5 | 0.204 | 0.129 | 0.165 | 3891 | 217 | | 5.28 |
| V015 | 1 | G1 | 2 | 0.695 | 0.185 | 0.721 | 1171 | 5876 | | 83.38 |
| V017 | 1 | G3 | 2.8 | 0.249 | 0.146 | 0.229 | 3552 | 220 | | 5.83 |
| V018 | 1 | G3 | 10 | 0.807 | 0.199 | 0.874 | 3130 | 28627 | | 90.14 |
| V021 | 1 | G2 | 0.5 | 0.876 | 0.141 | 0.922 | 985 | 30863 | | 96.91 |
| V026 | 1 | G2 | 7 | 0.864 | 0.150 | 0.918 | 232 | 5056 | | 95.61 |
| V029 | 0 | G1 | 7 | 0.153 | 0.143 | 0.110 | 789 | 28 | | 3.43 |
| V036 | 1 | G3 | 17 | 0.579 | 0.188 | 0.556 | 1960 | 3168 | | 61.78 |
| V041 | 1 | G2 | 5 | 0.685 | 0.182 | 0.712 | 1523 | 7269 | | 82.68 |
| V051 | 0 | G3 | 5 | 0.242 | 0.145 | 0.195 | 7176 | 662 | | 8.45 |
| V057 | 0 | G2 | 5.5 | 0.162 | 0.131 | 0.134 | 11369 | 436 | | 3.69 |
| V070 | 0 | G2 | 6 | 0.081 | 0.097 | 0.050 | 8955 | 95 | | 1.05 |
| V074 | 1 | G2 | 9 | 0.738 | 0.170 | 0.771 | 1486 | 12917 | | 89.68 |
| V077 | 0 | G1 | 1 | 0.273 | 0.183 | 0.221 | 1775 | 226 | | 11.29 |
| V085 | 1 | G2 | 3 | 0.583 | 0.226 | 0.584 | 4330 | 7830 | | 64.39 |
| V087 | 0 | G2 | 1.5 | 0.504 | 0.155 | 0.515 | 1186 | 1463 | | 55.23 |
| V089 | 1 | G2 | 14 | 0.609 | 0.176 | 0.602 | 1143 | 2756 | | 70.68 |
| V096 | 0 | G2 | 7 | 0.140 | 0.133 | 0.104 | 8206 | 285 | | 3.36 |
| V105 | 1 | G2 | 7 | 0.697 | 0.178 | 0.724 | 715 | 3617 | | 83.49 |
| V120 | 0 | G2 | 5 | 0.212 | 0.150 | 0.175 | 18007 | 1429 | | 7.35 |
| V1269 | 1 | G1 | 5 | 0.579 | 0.197 | 0.587 | 1386 | 2559 | | 64.87 |
| V162 | 0 | G2 | 8 | 0.219 | 0.161 | 0.184 | 8402 | 702 | | 7.71 |
| V168 | 1 | G2 | 6.3 | 0.682 | 0.157 | 0.688 | 503 | 2955 | | 85.45 |
| V174 | 1 | G2 | 14 | 0.719 | 0.173 | 0.758 | 3498 | 22093 | | 86.33 |
| V1958 | 0 | G1 | 6 | 0.607 | 0.184 | 0.634 | 4809 | 13429 | | 73.63 |
| V200 | 1 | G2 | 15 | 0.751 | 0.174 | 0.792 | 5282 | 52062 | | 90.79 |
| V206 | 0 | G2 | 1 | 0.472 | 0.139 | 0.463 | 166 | 101 | | 37.83 |
| V2110 | 0 | G2 | 4.5 | 0.531 | 0.227 | 0.567 | 1441 | 2008 | | 58.22 |
| V218 | 1 | G3 | 24 | 0.766 | 0.189 | 0.818 | 4732 | 39528 | | 89.31 |
| V224 | 1 | G3 | 17 | 0.413 | 0.244 | 0.361 | 1880 | 1007 | | 34.88 |
| V6023 | 1 | G2 | 22 | 0.667 | 0.187 | 0.695 | 12402 | 50440 | | 80.26 |
| V9006 | 0 | G2 | 6 | 0.120 | 0.114 | 0.089 | 15709 | 304 | | 1.9 |
| LN: lymph node (0 = negative; 1 = positive) | | | | | | | | |  |  |
| Grade: G1, well differentiated; G2, moderately differentiated; G3, poorly differentiated | | | | | | | | |  |  |
| DOI_mm: tumor depth of invasion | | | | | | | | |  |  |
| NRS: nodal risk score | | | | | | | | |  |  |
| cell_negative: number of cells with NRS less than 0.5 | | | | | | | | |  |  |
| cell_positive: number of cells with NRS greater or equal to 0.5 | | | | | | | | |  |  |
| Positive%: percentage of cells with NRS greater or equal to 0.5 | | | | | | | | |  |  |
